# On Blistering in Gleet

**Published:** 1852-08

**Authors:** John L. Milton


*On Blistering in Gleet.* By John L. Milton, Esq.—Not merely will a single blister frequently cure the most prolonged gleet—not merely will it rapidly sweep away all dregs of the disease in its ordinary course, but it will often cure those runnings, which have resisted all known and used methods. I have seen two blisters, with a mild injection or two, at once cure a clap which had defied the most energetic treatment; and *as I never found a case which resisted blistering and injections together, that was not complicated with strictvre, or affection of testicle,* I am slowly arriving at the conviction, *that every case of clap or gleet, however obstinate, may, if uncomplicated, be cured by blistering, singly or combined.*

To illustrate and urge forward this operation by every means in my power—to invite attention to it, that it may be put to the severe test of practice, to attest it by cases which I have collected and watched—to point out the necessity of quitting the beaten paths of treatment, and try a new remedy, more powerful than those in use, is the object of this paper. But as so many remedies have fallen into disuse from the indiscriminate use or misapplication of them, and as so many which are in favor have been arrested on their path by obstacles issuing from the same sources, I must here enter my protest against being supposed to recommend blistering in gleet, unless properly applied, and in the cases I have referred to.

In order that a blister be properly applied, there are some points, which, however trifling they may seem, require as much attention as the leading features of the case,

Where these are neglected, blistering is apt to produce such a filthy excoriated mass, that the patient will not submit to it a second time; whereas, if carefully laid on and dressed, it is, from its being out of the reach of friction, in the ordinary movements of the body, even less troublesome than if on a limb, or the trunk. Before putting it on, the hair at the root of the penis is cut off, and, if the foreskin be naturally retracted, it must be drawn a little forward over the glans. A piece of paper is then to be fitted on the penis, and cut till it exactly covers it, from the root to within half an inch of the mouth of the urethra. This is then laid down on the blister, which is cut out by it, wrapped round the penis, and fastened with threads behind the glans and near the root. The patient should remain perfectly quiet during the time it is on, lest any motion should bring the blister against the scrotum, and vesicate it; but he must not apply it on going to bed, or he will most likely fall asleep, and not awake till the penis is one mass of vesications—a state productive of an unnecessary amount of suffering.

In the milder cases, or where the skin is tender, an hour, or an hour and a half, will be sufficient. The blister is then removed; if there are any vesicated spots, they are covered with pieces of linen spread with zinc ointment, and then a layer of cotton is bound over these, and covered with a piece of linen, kept on by a thread, or, what is better, two very thin rings of vulcanized India rubber.

Where a severer case renders a more energetic employment of the remedy necessary, it must be kept on two or four hours, until free vesication is produced; zinc ointment is then applied.

To protect the penis from friction, a T bandage, with a linen bag sown into the part which receives the penis, or a handkerchief carried round the waist, and dipping in front, so as to receive the penis, and keep it up against the abdomen, is necessary.

The first effect of this application is, to increase the discharge considerably, which then terminates, either by altering its character, becoming ropy and mucous, and finally disappearing in a few days, or by remaining somewhat more persistent, and requiring a few injections, when the penis is so far advanced towards healing, that it can be handled without pain, or demanding even a second blister. One of the most cleanly and convenient, and least painful forms of blister, is Brown's blistering tissue; it causes much less irritation, and heals much more quickly than the ordinary blister. The blistering fluids, if strong enough to vesicate, caused such pain, that I soon renounced the use of them.

How does this remedy act. By counter-irritation, will, perhaps, be the answer. But if this were the case, why should there be increased action in the urethra for a few days and why should the discharge from the urethra begin to disappear, when the counter irritant-surface is healing up? It would seem as if the organized constituents of the urethra are capable of keeping up a certain amount of over-action for an indefinite time; but that when hurried beyond this by a healthy stimulant, *a rebound* takes place, which leaves them less capable than before of furnishing a secretion, morbid in amount or in quality, or in both. We see something similar in prurigo pubis, where a blister causes an exacerbation of the symptoms, succeeded, however, in some cases, by a healthier state of the skin; in bubo, treated by blister, etc.—*Braithwaite's Retrospect.*

